# Effects of coffee and caffeine anhydrous on strength and sprint performance

**DOI:** 10.1186/1550-2783-12-S1-P57

**Published:** 2015-09-21

**Authors:** Eric T Trexler, Erica J Roelofs, Katie R Hirsch, Meredith G Mock, Abbie E Smith-Ryan

**Affiliations:** 1 Department of Exercise and Sport Science, University of North Carolina at Chapel Hill, Chapel Hill, NC, USA

## Background

Caffeine is a commonly used ergogenic aid and is included in many pre-workout formulations marketed towards athletes engaged in high-intensity exercise. Previous studies have directly compared the effects of coffee (COF) and anhydrous caffeine (CAF) on endurance performance, with equivocal results reported. To our knowledge, COF and CAF have not yet been directly compared in the context of strength and sprint performance. The purpose of the current randomized, double-blind study was to compare the effects of acute COF and CAF intake on strength and sprint performance.

## Methods

Fifty-four resistance-trained male participants (mean ± SD; age = 20.1 ± 2.1 yrs; height = 177.3 ± 5.6cm; weight = 78.8 ± 8.8 kg; habitual caffeine intake = 32.9 ± 59.6mg/day) completed baseline strength testing, consisting of both one-rep max (1RM) and repetitions to fatigue (RTF) for leg press (LP) and bench press (BP). Following strength testing, a friction-loaded cycle ergometer was loaded with a resistance of 95g/kg of bodyweight and participants completed a repeated sprint protocol consisting of five, ten-second sprints separated by one minute of passive rest. Peak power (PP) and total work (TW) were recorded for each sprint, along with average PP and TW values for the entire protocol (all five sprints). At least 48 hours later, participants returned for post-testing and ingested a beverage containing either CAF (300mg), a caffeine-matched dose of instant COF (8.9g, yielding 303mg of caffeine), or a flavored placebo (PLA) 30 minutes prior to exercise. Prior to each visit, participants were instructed to maintain similar dietary habits, abstain from strenuous exercise for at least 24 hours, and avoid caffeine intake for at least 48 hours. Change scores were compared using one-way ANOVAs, and 95% confidence intervals (mean ± 1.96 × SEM) were constructed for each dependent variable.

## Results

Leg press 1RM was improved more by COF compared to CAF (Δ = 32.2 ± 18.6 vs 15.3 ± 16.9lb, p = 0.04), but not to PLA (p = 0.99). Significant interactions were not observed for BP 1RM, BP RTF, or LP RTF (p > 0.05). There were no significant sprint × treatment interactions for changes in PP or TW (p > 0.05). For TW, a main effect for sprint was observed (p = 0.02). 95% confidence intervals revealed a significant improvement in sprint 1 TW for CAF [81.4, 623.9J], but not COF [-121.0, 376.2J] or PLA [-239.9, 180.1J]. Reductions were observed in sprint 4 PP [-64.9, -2.5W], sprint 2 TW [-321.2, -66.1J], sprint 4 TW [-403.1, -57.6J], and average TW [-219.0, -40.2J] in PLA, but not in CAF or COF.

## Conclusion

Neither COF nor CAF improved strength outcomes to a greater degree than PLA. Repeated sprint results suggest that both COF and CAF attenuated power reductions to a similar degree. Considering the potential health benefits associated with regular COF consumption, COF may be considered a suitable source of pre-exercise caffeine for high-intensity exercise.

